# A *Chlamydomonas*-Derived Human Papillomavirus 16 E7 Vaccine Induces Specific Tumor Protection

**DOI:** 10.1371/journal.pone.0061473

**Published:** 2013-04-23

**Authors:** Olivia C. Demurtas, Silvia Massa, Paola Ferrante, Aldo Venuti, Rosella Franconi, Giovanni Giuliano

**Affiliations:** 1 ENEA, Italian National Agency for New Technologies, Energy and Sustainable Economic Development, Casaccia Research Center, Rome, Italy; 2 Ylichron S.r.l., ENEA Casaccia Research Center, Rome, Italy; 3 Laboratory of Virology, Regina Elena National Cancer Institute, Rome, Italy; Nanyang Technological University, Singapore

## Abstract

**Background:**

The E7 protein of the Human Papillomavirus (HPV) type 16, being involved in malignant cellular transformation, represents a key antigen for developing therapeutic vaccines against HPV-related lesions and cancers. Recombinant production of this vaccine antigen in an active form and in compliance with good manufacturing practices (GMP) plays a crucial role for developing effective vaccines. E7-based therapeutic vaccines produced in plants have been shown to be active in tumor regression and protection in pre-clinical models. However, some drawbacks of in whole-plant vaccine production encouraged us to explore the production of the E7-based therapeutic vaccine in *Chlamydomonas reinhardtii*, an organism easy to grow and transform and fully amenable to GMP guidelines.

**Methodology/Principal Findings:**

An expression cassette encoding E7GGG, a mutated, attenuated form of the E7 oncoprotein, alone or as a fusion with affinity tags (His6 or FLAG), under the control of the *C. reinhardtii* chloroplast *psbD* 5′ UTR and the *psbA* 3′ UTR, was introduced into the *C. reinhardtii* chloroplast genome by homologous recombination. The protein was mostly soluble and reached 0.12% of total soluble proteins. Affinity purification was optimized and performed for both tagged forms. Induction of specific anti-E7 IgGs and E7-specific T-cell proliferation were detected in C57BL/6 mice vaccinated with total *Chlamydomonas* extract and with affinity-purified protein. High levels of tumor protection were achieved after challenge with a tumor cell line expressing the E7 protein.

**Conclusions:**

The *C. reinhardtii* chloroplast is a suitable expression system for the production of the E7GGG protein, in a soluble, immunogenic form. The production in contained and sterile conditions highlights the potential of microalgae as alternative platforms for the production of vaccines for human uses.

## Introduction

Plant molecular pharming represents a well-established biotechnology area that includes the production of protein biopharmaceuticals such as enzymes, hormones, antibodies, and vaccine antigens in plant systems. Plant-produced proteins represent a significant fraction of pharmaceuticals in advanced preclinical and clinical trial status [1], [2]. However, plant platforms present some drawbacks, including long time to generating stable transgenic lines, non homogeneous protein production in different tissues, impact of pests and diseases even in controlled conditions (greenhouses) and, more importantly, growth in non-sterile conditions that make difficult the application of good manufacturing practices (GMP) necessary for the production of pharmaceuticals. To circumvent some of these drawbacks, transient expression [3] or *in vitro* culture [4] have emerged as alternative platforms. FDA has recently approved the first plant-made drug for human use, an enzyme produced in genetically engineered carrot cells for treating type 1 Gaucher's disease [5].

Microalgae have been proposed as an alternative molecular pharming system. This relatively new platform offers several advantages, including: 1) short time from transformation to scaling up; 2) rapid growth (doubling time of few hours) and ease of cultivation; 3) safety, because microalgae do not harbor human pathogens, many are Generally Regarded As Safe (GRAS) organisms, and grow in axenic conditions facilitating production of biopharmaceuticals in GMP conditions; 4) homogeneity of protein production with the use of controlled bioreactors. In particular, the green unicellular alga *Chlamydomonas reinhardtii* has emerged as a model system, with its three genomes (nuclear, plastidial and mitochondrial) completely sequenced [6], and the easy generation of stable transgenic or transplastomic lines in few weeks [7], [8]. While expression from the nuclear genome is subject to position effects [9] and gene silencing [10], expression from the chloroplast genome is well established [11], [12]. Like bacteria, the chloroplast lacks the machinery to perform complex post-translational modifications such as glycosylation (the glycosylated proteins come from the Endoplasmic Reticulum), but, unlike *E. coli*, the *Chlamydomonas* chloroplast allows the formation of disulfide bonds and is able to perform some types of phosphorylation. In addition, it contains low protease levels as well as several molecular chaperones aiding protein folding [11]. Unlike higher plants, that present several hundreds of chloroplasts per cell, each with up to 100 genome copies, *C. reinhardtii* has a single chloroplast, with about 80 genome copies. Consequently, conversion of all copies of the chloroplast genome to the recombinant form (homoplasmy) is facilitated. *C. reinhardtii* has been used for the expression of recombinant vaccines [13]–[15], fully functional antibodies [16], [17], therapeutics [18] and other proteins of biotechnological relevance [19], with yields ranging from undetectable levels to about 5% of Total Soluble Proteins (TSP) [8]. A comparative work done with 7 different therapeutic proteins expressed in the *C. reinhardtii* chloroplast demonstrates the high variability in expression levels, indicating that protein yields depend primarily on the intrinsic properties of each protein expressed [18]. Studies on chloroplast gene expression and regulation in *C. reinhardtii* for molecular pharming are still in their infancy: the first biopharmaceutical was expressed in 2003 [16] and a large knowledge and technology gap needs to be filled in order to bring microalgal productivity to a level similar to that of plants or other well established platforms.

High risk HPVs (hr-HPVs) are responsible for 6.1% of total cancer cases worldwide [20] and represent the etiological agents of virtually all (99.7%) cervical cancers (CC) [21] with the HPV16 genotype accounting for more than 50% of these cases [22]. The current approved prophylactic vaccines for HPV (GARDASIL®, Merck and CERVARIX®, GlaxoSmithKline) are expensive, not able to protect already-infected people (more than 30 million people worldwide) and are not useful for treating established lesions and tumors. For these reasons, and also because conventional therapies for CC are not completely effective in eradicating the tumor and are usually invasive, toxic, and associated with 10–20% recurrence, alternative CC treatments such as therapeutic vaccines are extremely important. The E7 oncoprotein of hr-HPVs is involved in malignant cellular transformation and represents an ideal antigen for the development of therapeutic vaccines [23]. Currently, commercial therapeutic vaccines against HPV-induced cancers are not available, but different strategies based on the *E7* oncogene have been explored, with some cases currently in clinical trial [24]. In any case, the production of therapeutic vaccines against cervical cancer still remains an open research field and a successful therapeutic formulation has to satisfy requirements like safety, low cost and ability to overcome immuno-suppression at the tumor site in order to effectively stimulate cell-mediated responses.

Recombinant E7-based vaccines have been produced in plant systems [25]. Consistent protein yield have been reached only when the E7 protein was expressed as fusion with a stable protein carrier such as bacterial lichenase [26], while relatively low levels were obtained for the non-fused E7 protein expressed transiently [27] or via chloroplast transformation [28]. Despite the demonstration of the effectiveness of E7-based, plant-produced therapeutic vaccines in pre-clinical models [26], [27], [29], [30], clinical trials with plant-derived E7-based vaccines are lagging due to difficulties in production in GMP conditions. In the present work we report the production of an immunogenic, soluble E7 protein in *C. reinhardtii* and demonstrate its effectiveness in protecting against cancer development in a pre-clinical model.

## Results and Discussion

### Transformation of the *Chlamydomonas* chloroplast with a mutated form (*E7GGG*) of the HPV16 *E7* oncogene

**Figure 1:**
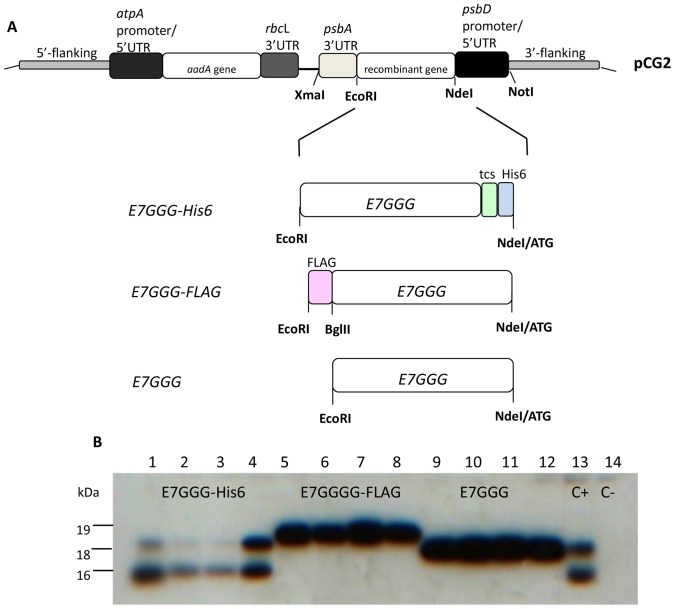
Chloroplast transformation of *C. reinhardtii* with recombinant E7 genes. **A.** Map of the pCG2 chloroplast transformation vector. E7GGG gene variants are placed under the control of the *psbD* promoter/5′UTR and the *psbA* terminator/3′UTR (expression cassette), while the selectable *aadA* marker is placed under the control of the *atpA* promoter/5′UTR and the *rbcL* terminator/3′UTR (resistance cassette). Regions indicated as 5′ and 3′ flanking correspond to regions where homologous recombination between the vector and the chloroplast genome occurs (see Figure S2). His6 = Histidine hexapeptide; tcs = thrombin cleavage site; FLAG = Flag affinity tag (Figure S1). **B.** Western blot of total proteins from 2×10^6^ cells of the four highest-expressing homoplasmic transformants for each E7GGG variant. E7GGG-FLAG (lanes 5–8) and E7GGG (lanes 9–12) proteins migrate as single bands of about 19 and 18 kDa, respectively, E7GGG-His6 (lanes 1–4, 13) migrates as a doublet of 16 kDa and 18.5 kDa. C+ = transformant expressing E7GGG-His6. C− = transformant obtained with the pCG1 vector (pCG2 vector without the expression cassette).

To avoid safety concerns associated with the administration of an oncoprotein in humans, we expressed an attenuated (mutated) form of the E7 protein, named E7GGG, lacking the pRb interaction ability that causes oncogenic transformation of mammalian cells [31]. We expressed E7GGG itself and two affinity tag fusions, E7GGG-FLAG [32] and E7GGG-His6 [33]. The corresponding coding sequences were codon-optimized for chloroplast expression [34] and placed under the control of the *psbD* promoter and 5′UTR and *psbA* terminator and 3′UTR (expression cassette) (Figure S1). The chloroplast transformation vector pCG2 (Figure 1A) was obtained by insertion of the expression cassette in the pCG1 vector, which also contained a spectinomycin-resistance cassette and two fragments of the *psaA* intron (5′ and 3′ flanking in Figure 1A) that mediate insertion in the chloroplast genome by homologous recombination.

All constructs were introduced in the cell wall-less (*cw15*) mutant strain of *C. reinhardtii* by transformation with glass beads [35]. Chloroplast transformants were selected on TAP+agar plates containing 100 µg/ml spectinomycin and screened by PCR for integration in the chloroplast genome (Figure S2). After at least 10 rounds of streaking for single colonies in selective medium, transformants were also screened for homoplasmy. For all three constructs, transgenes resulted stably integrated in the chloroplast genome and homoplasmic cell lines were obtained (Figure S2).

### Production and purification of soluble E7GGG protein variants

For each construct (Figure 1A), 40 independent transformants were brought to homoplasmy and screened through Western blotting. All three protein variants were expressed, at different levels, in 85–95% of the analyzed transformants. The four best producers for each E7GGG variant were compared to evaluate possible influences of the affinity tag on maximum accumulation of the E7GGG protein. Higher levels were obtained for E7GGG and E7GGG-FLAG than for E7GGG-His6 (Figure 1B). An inhibitory effect of the His6 tag on chloroplast expression has been observed by others (S. Mayfield, personal communication). This inhibitory effect may be due to impairment of a chloroplast function by the His6 peptide, as suggested by the fact that the best E7GGG-His6 expressor shows almost 50% inhibition of cell growth (Figure S3). In spite of this inhibition, we were able to obtain measurable levels of soluble E7GGG-His6 in *Chlamydomonas* chloroplasts, while we have been unsuccessful in obtaining the same protein variant by Potato Virus X (PVX)-mediated transient expression in *Nicotiana benthamiana* leaves (data not shown).

**Figure 2:**
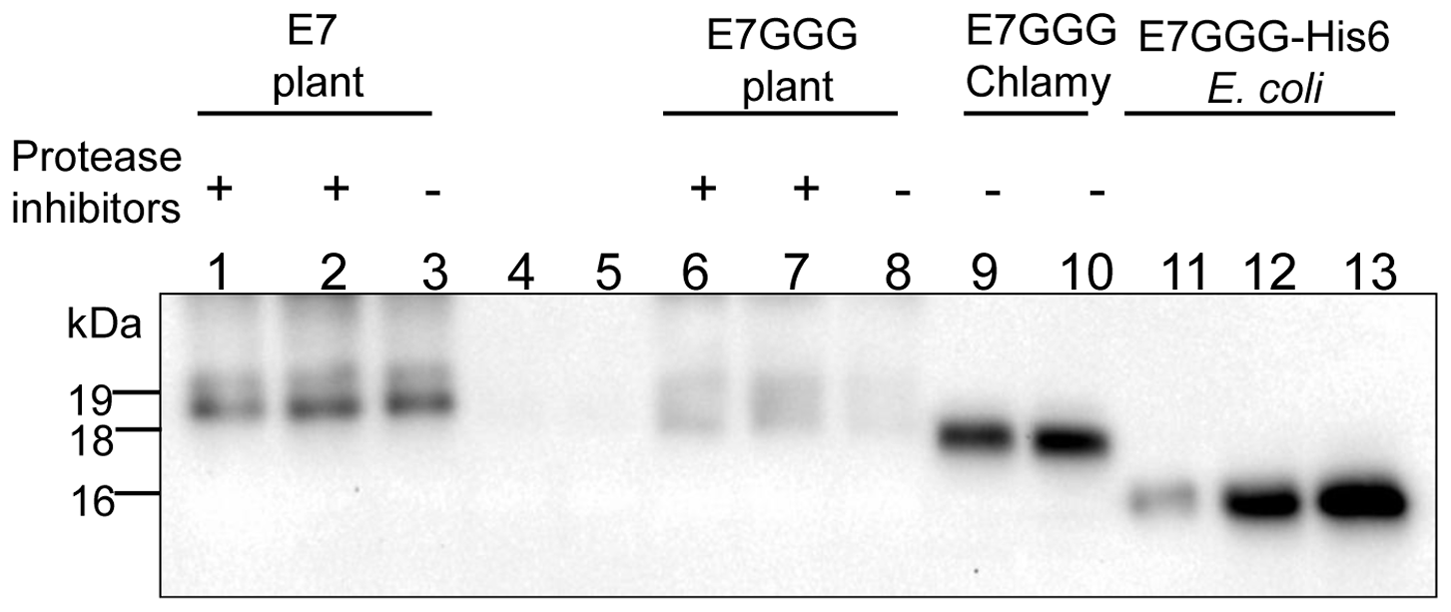
Comparison of plant- and algae-produced E7 proteins. E7 and E7GGG amounts in soluble extracts from *N. benthamiana* leaves infected with PVX or from transplastomic *C. reinhardtii* (E7GGG). Protein extraction was performed with PBS buffer (21 mM Na_2_HPO_4_, 2.1 mM NaH_2_PO_4_, 150 mM NaCl, pH 7.2). For each sample, 10 µg of TSP were loaded on SDS-PAGE. Protein expression levels were determined by Western blotting, comparing the intensity of the E7 and E7GGG bands with different amounts of E7GGG-His6 purified from *E. coli*. Lanes 1, 2: two independent extractions of plant-derived E7 in the presence of protease inhibitors; lane 3: plant E7 extracted without protease inhibitors; lanes 4, 5: empty lanes; lanes 6, 7: two independent extractions of the plant produced E7GGG protein in the presence of protease inhibitors; lane 8: sample of plant produced E7GGG protein extracted without protease inhibitors; lanes 9, 10: two independent extractions of the *Chlamydomonas*-produced E7GGG protein extracted without protease inhibitors; lanes 11–13: E7GGG-His6 purified from *E. coli* (5, 10 and 20 ng, respectively).

We compared the amounts of E7 and E7GGG proteins produced in *N. benthamiana* by PVX-infection [27] to those of E7GGG from *C. reinhardtii*. Both forms produced in *N. benthamiana* were present at lower concentrations with respect to the E7GGG protein produced in *Chlamydomonas* (Figure 2). Additionally, the attenuated E7GGG form produced in *N. benthamiana* shows some instability in the absence of protease inhibitors. We also tested the extractability, using different buffers, of E7GGG produced in *N. benthamiana* and in the *Chlamydomonas* chloroplast. In all buffers, the plant-expressed protein was mostly found in the insoluble fraction, while the *Chlamydomonas*-expressed one was highly soluble (Figure S4).

**Figure 3:**
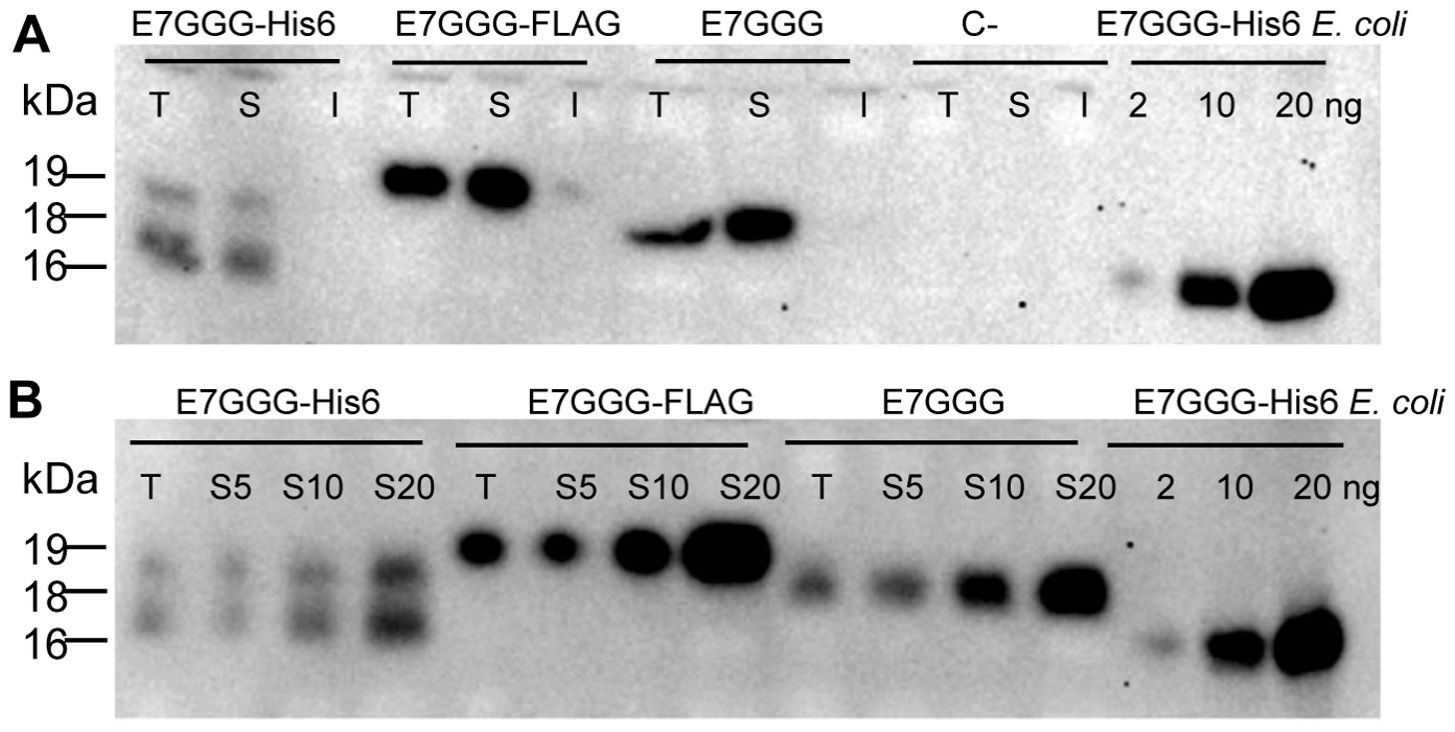
Solubility and quantification of E7GGG proteins. **A.** Western blot of total (T), soluble (S) and insoluble (I) protein fractions of the highest-expressing transformant of each E7GGG variant, normalized for TSP content (10 µg). Protein extraction was performed with 100 mM Tris-HCl, 200 mM sucrose pH 7.5 and total, soluble and insoluble fractions obtained from 2×10^5^ cells were loaded on a gel to assay the solubility of each E7GGG protein variant. Similar results were obtained using PBS as extraction buffer. C− = transformant obtained with the pCG1 vector. **B.** Western blot with increasing amounts of TSP from the highest-expressing transformant for each E7GGG variant. Protein extraction was performed with the same buffers described in panel A. T = 10 µg total proteins. S5, S10, S20 = 5, 10, 20 µg of soluble proteins. In both panels, quantification was performed using known amounts (2, 10 and 20 ng) of E7GGG-His6 protein purified from *E. coli*.

All three protein variants expressed in *Chlamydomonas* accumulated almost exclusively in the soluble cellular fraction (Figure 3A). Protein quantification was performed by immunoblotting, using different amounts of E7GGG-His6 protein purified from *E. coli* as a standard (Figure 3B). The maximum protein yields were about 0.02% TSP for E7GGG-His6, 0.1% for E7GGG and 0.12% for E7GGG-FLAG. These yields are coherent with results obtained with other proteins expressed in the *Chlamydomonas* chloroplast [8]. A similar yield (0.1% TSP) was obtained for the E7 protein in transplastomic tobacco plants [28].

**Figure 4:**
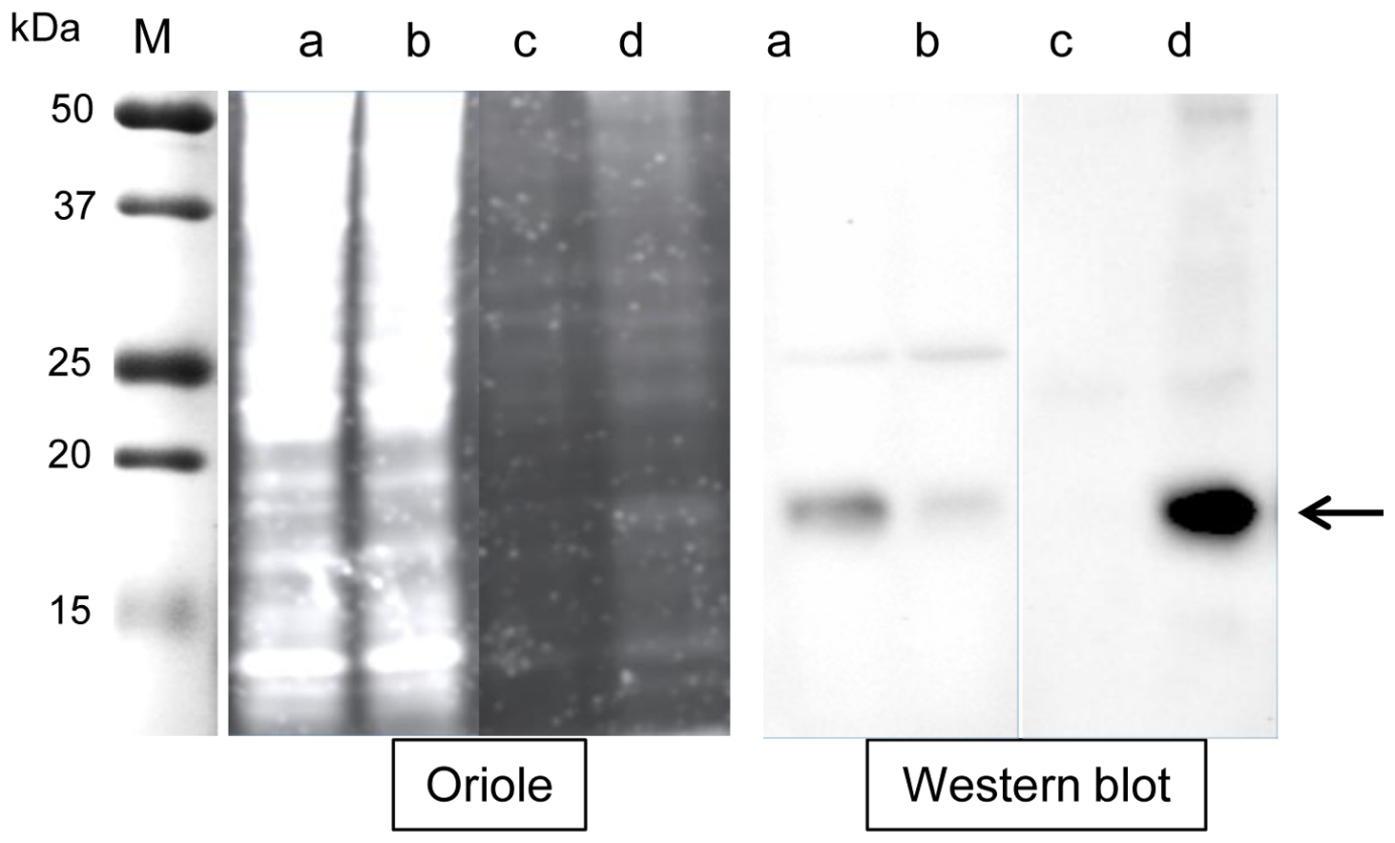
Affinity purification of the E7GGG-FLAG protein. Oriole-stained gel and Western blot of 10 µl of the following samples, a: E7GGG-FLAG soluble extract before affinity purification; b: column flow-through; c: column wash fraction; d: fraction eluted with 1 M Arg-HCl pH 3.5. M = molecular weight marker.

Up to now, purification of the E7 or E7GGG proteins from plants has been described only for fusion forms with carrier polypeptides such as bacterial lichenase [26], [36], or HPV L1 and E6 proteins [37]. We purified the E7GGG- FLAG protein by affinity chromatography on anti-FLAG M2 affinity resin (see Materials and Methods). Good protein recovery was obtained when using 1M Arg-HCl pH 3.5 as elution buffer (Figure S5) [38]. The eluted protein is detectable using the Oriole fluorescent stain (Bio-Rad) (Figure 4). After dialysis against PBS 1X+0.1 mM ZnSO_4_ and concentration, about 70% of the original protein was recovered with a final yield of about 7 µg of purified E7GGG-FLAG protein/liter of *Chlamydomonas* culture.

Purification of the E7GGG-His6 protein was performed using the Ni-NTA resin, with a yield of 1 µg of protein/liter of *Chlamydomonas* culture. Both in the crude extract or as purified protein, E7GGG-His6 is present as two bands with different apparent molecular weight (MW) of 16 kDa and 18.5 kDa. The two bands were present also after the addition of 10 mM 2-mercaptoethanol and 10 mM dithiothreitol (DTT) and boiling for more than 10′ (Figure S6A). Upon separation on 15% SDS-PAGE, a third band was observed, which increased after calf intestinal phosphatase (CIP) treatment (Figure S6B). This result suggests that the two fastest-migrating bands represent phosphorylated forms of the protein.

### Induction of immune responses and protection of mice against E7-expressing tumors by the E7GGG vaccine

**Figure 5:**
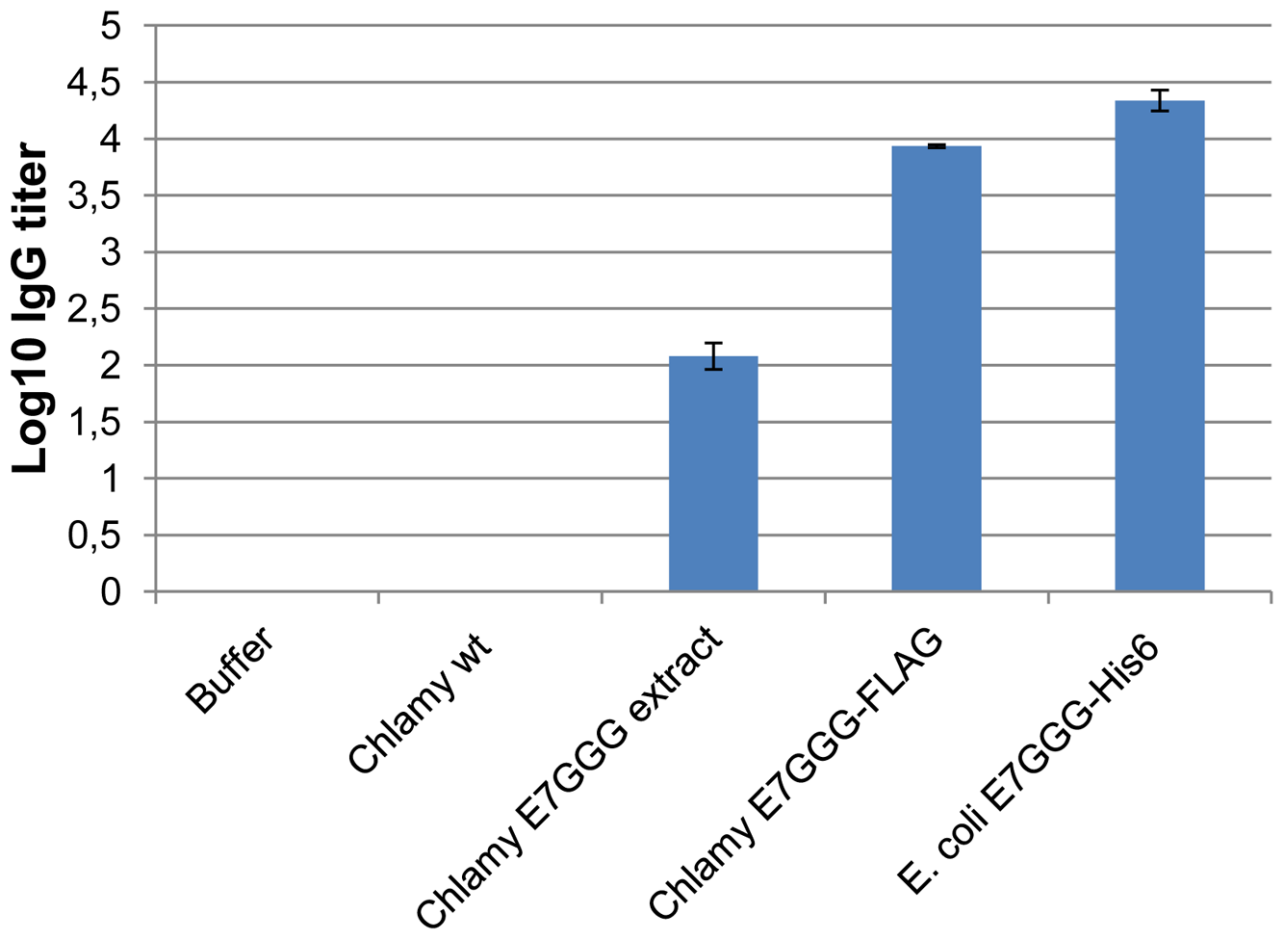
E7-specific serum IgG responses. Sera from vaccinated mice after the fourth boost. The ELISA antibody titers were calculated as the log_10_ of the reciprocal antibody dilution that showed an OD_405_ value above the cut-off value, which was defined as the average OD_405_ value of non-immunized sera+3 standard deviations. Data are representative of three technical replicates using as coating antigen 100 ng/well of the E7-His6 protein from *E. coli* diluted in PBS+0.1 mM ZnSO_4_ buffer. Similar results were obtained when coating with bicarbonate buffer.

Preliminary experiments had already shown that the *Chlamydomonas* extract (both in the absence or presence of the E7GGG protein) had no toxic effect on mice when injected sub-cutaneously. Therefore, groups of 8 C57BL/6 mice were immunized 5 times, at 2-week intervals, by sub-cutaneous administration of the following preparations (in the presence of 10 µg/mouse of QuilA adjuvant): (i) soluble algal extract containing the E7GGG protein (1 mg of TSP containing 1 µg of E7GGG/mouse); (ii) purified E7GGG-FLAG protein from *Chlamydomonas* (2 µg of protein/mouse); (iii) purified E7GGG-His6 from *E. coli* (2 µg of protein/mouse). As negative controls, mice were vaccinated with either buffer alone, or with *C. reinhardtii* extract devoid of E7GGG. Both E7GGG-His6 purified from *E. coli* and E7GGG-FLAG purified from *Chlamydomonas* induced high titers of specific IgGs after the fourth boost, while the *Chlamydomonas* E7GGG-containing extract showed a much lower IgG induction (Figure 5). Insignificant IgG induction was detected in the two control groups.

**Figure 6:**
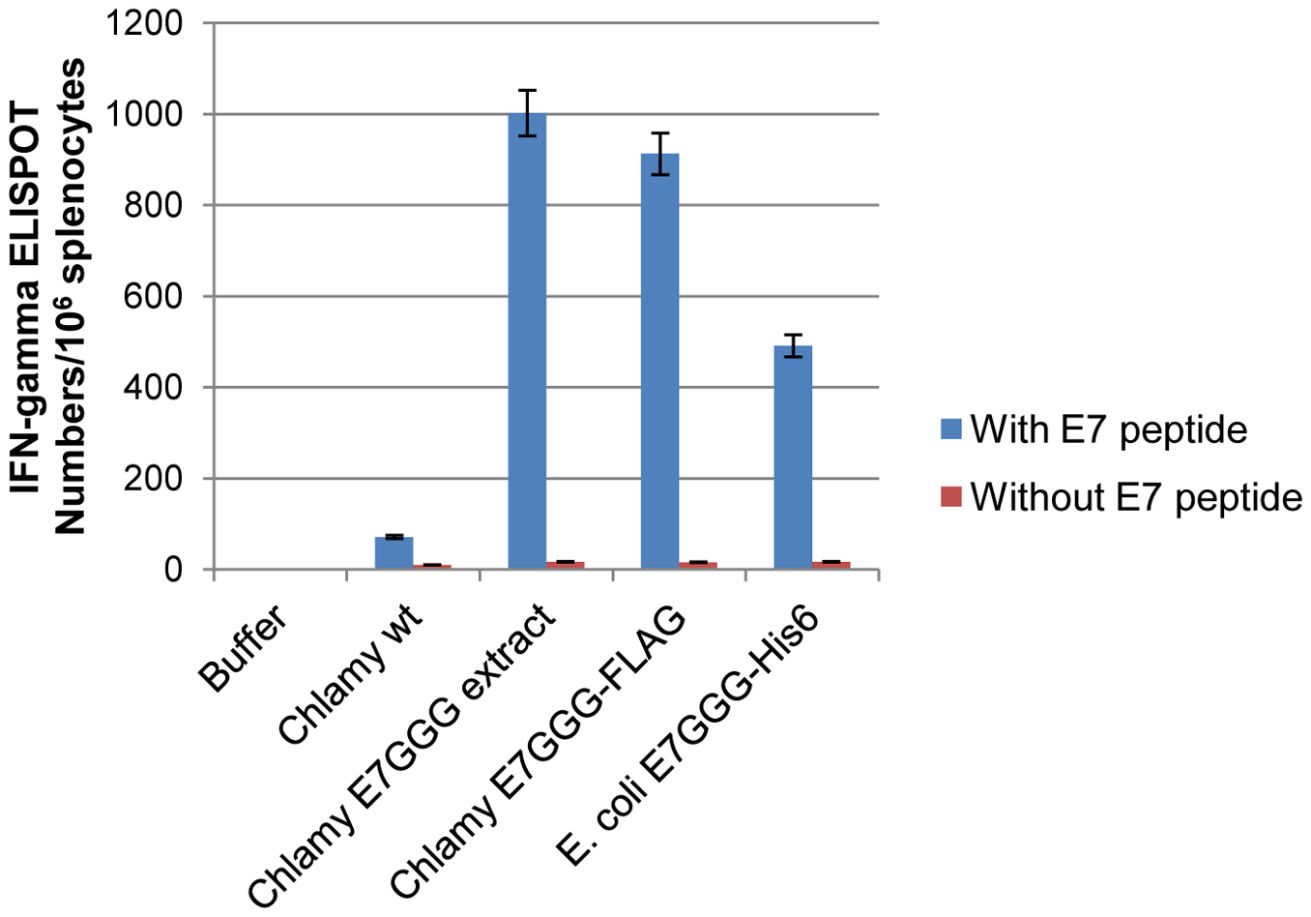
ELISPOT analysis of splenocytes of vaccinated mice. Splenocytes were recovered from sacrificed animals after the last boost and stimulated with 1 µg/ml of specific CTL E7 peptide (amino acids 49-57, RAHYNIVTF) (blue bars) or not stimulated (red bars). The number of IFN-γ producing E7-specific T-cell precursors was determined using an anti-IFN-γ antibody. Data are presented as mean number of spots per 10^6^ splenocytes. Error bars represent standard deviation of three technical replicates.

Since humoral immune responses play a marginal role in anti-cancer responses, while the induction of E7-specific cell-mediated (in particular CD8^+^ T cells) immune response is generally correlated to anti-cancer activity [39], we investigated the presence of the latter by Enzyme-Linked Immunosorbent Spot (ELISPOT) assay. Higher numbers of IFN-γ-secreting cells were detected in mice vaccinated with the E7GGG protein from *C. reinhardtii*, both in crude extracts and as purified protein, compared to the group vaccinated with *E. coli* E7GGG-His6 (Figure 6). No, or very few, IFN-γ-secreting cells were detected in the control groups.

Cell-mediated immune responses were also evaluated by measuring the Delayed-Type Hypersensitivity (DTH) response (that represents antigen-specific cytokine mediated inflammation involving Th1 type cytokines) [40] to the HPV16 E7 protein in vaccinated mice, before challenge with the TC-1 cells. An E7-specific DTH response was observed in mice vaccinated with both the *Chlamydomonas* E7GGG-FLAG antigen and with the *Chlamydomonas* E7GGG-containing extract (Table 1). In the latter case, the response exceeded that recorded in the group vaccinated with the *E. coli* E7GGG-His6 antigen. Mice immunized with either buffer or *Chlamydomonas* wt extract showed no inflammatory response.

**Table 1:** Delayed type hypersensitivity to E7 protein in vaccinated mice.

	Δ ear thickness^a^ ± S.D. at 48 h
Buffer	1.0±1.1
Chlamy wt	1.8±1.6
Chlamy E7GGG-containing extract	19.3±2.5
Chlamy E7GGG-FLAG	9.5±2.0
E. coli E7GGG-His6	13.5±2.6

S.D., standard deviation.

a Ear swelling was reported as the mean of the differences (Δ) in thickness between challenged and unchallenged control ears from five mice per group (mm ear thickening ×10^−2^).

**Figure 7:**
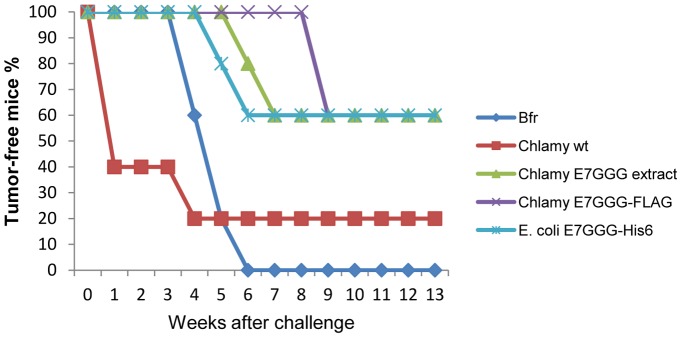
Mouse protection against TC-1 -induced tumor. Two weeks after the last boost, five vaccinated mice for each group were challenged by subcutaneous injection of 5×10^4^ TC-1 cells/mouse. The presence of the tumor was monitored by palpation twice a week. Data are represented as percentage of tumor-free mice.

Tumor protection was evaluated by challenging vaccinated mice with 5×10^4^ cells from the TC-1 tumor cell line, expressing the E7 antigen, two weeks after the last injection [26], [27], [29], [41]. Final data collected 13 weeks after the challenge showed that *Chlamydomonas* crude extract containing E7GGG, as well as purified E7GGG-FLAG from *Chlamydomonas* and E7GGG-His6 from *E. coli* elicited tumor protection in 60% of mice (Figure 7). The group of mice vaccinated with the *Chlamydomonas* E7GGG-FLAG purified protein remained tumor-free for a longer time (100% tumor-free mice after 9 weeks) than the other two groups. Insignificant protection was observed in the two control groups. Taken together, our data indicate that the microalga-produced E7GGG protein is highly immunogenic, both in crude extracts and in purified form, providing effective tumor protection in a preclinical system.

## Conclusions

Since biopharmaceuticals have become increasingly important for the treatment of various human diseases, it is very important to improve existing expression systems as well as to develop new ones. In this context, the benefits arising from molecular pharming and, in particular, from the use of microalgae as production platforms (i.e. ease of scale-up, GMP production, lower production costs), represent a highly attractive perspective.

Important clinical successes have been obtained on patients with HPV16-induced precancerous lesions, treated with vaccines comprising HPV16 E6 and/or E7 proteins [42]–[44]. Here, we show that an attenuated form of the HPV E7 protein can be produced in the *Chlamydomonas* chloroplast in a highly soluble form, affording cancer protection in a preclinical animal model. Although the amounts of protein produced are still insufficient for clinical experimentation, the present work shows the possibility of using microalgae for the production of bio-active HPV E7 antigen. Additionally, the obtainment of soluble, purified E7 protein from microalgae offers the possibility to perform detailed biochemical and chemical/physical studies, aimed at verifying its structure and biological activity, which up to now have been performed only on proteins expressed in bacterial systems [45], [46]. Future developments on gene expression optimization (i.e. the use of different promoters and/or the integration of the transgene in more suitable regions of the chloroplast genome) could lead to increased protein yields, needed for clinical studies.

## Materials and Methods

### Ethics statement

Animal experiments performed in this study were conducted according to the Institutional animal use guidelines and the Italian law DL 116/92. All procedures for animal experiments were approved by the Ethical Committee for Animal Experimentation of the Istituti Fisioterapici Ospitalieri (IFO) at the Regina Elena National Cancer Institute. Mice were anesthetized with 40–50 mg of Zoletil (tiletamine+zolazepam Virbac, Milan, Italy) per 1 kg of body weight and all efforts were made to minimize suffering.

### Synthesis of HPV16 E7 gene variants

We used the mutated form of the HPV16 E7 protein (NP_041326) named E7GGG, firstly realized for genetic vaccination by Smahel and colleagues [31]: it presents the mutated GLYGYG amino acid sequence instead to the native DLYCYE (amino acids 21–26) motif. The E7GGG protein was fused to two purification tags generating two variants: E7GGG-His6 (carrying the hexa-histidine tag and the thrombin cleavage site fused to the N-terminus); and E7GGG-FLAG (carrying the DYKDDDDKS FLAG tag [18], fused to the C-terminus). Codon optimization was performed using the Optimizer application (http://genomes.urv.es/OPTIMIZER) as described [34], aiming at a CAI (“Codon Adaptation Index”) value of 0.8. The optimized nucleotide sequence was synthesized by Genscript Co., USA. Restriction sites NdeI and EcoRI were inserted at the 5′ and 3′, respectively, of the gene, in order to subsequently re-place the gene with the other E7GGG variants, and restriction sites XmaI and NotI were placed at the 5′ and 3′, respectively, of the expression cassette, to introduce it into the chloroplast transformation vector pCG1 (kindly provided by Prof. J. D. Rochaix, University of Geneve). To obtain the *E7GGG* gene, the *E7GGG-His6* sequence was amplified with the forward primer 5′-TTACATCAT**ATG**CACGGTGATACGCCTACATT-3′ (the NdeI site is underlined and the start codon is in bold) and the reverse primer 5′-TTGAATTCTAGA**TTA**AGGTTTTTGTGAACAAATAG-3′ (the EcoRI site is underlined and the stop codon is in bold). To obtain the *E7GGG-FLAG* gene we created a pCG2 vector harboring the FLAG-tag (indicated as pCG2-FLAG) followed by a stop codon upstream the *psbA* terminator by annealing the following primers and cloning them in the NdeI and EcoRI sites of pCG2: 5′-*TA* TGATTCGAAGATCTgactataaagatgatgacgataaatca**TAA** G-3′ and 5′-ACTAAGCTTCTAGActgatatttctactactgctatttagt**ATT** C*TTAA*-3′ (NdeI, EcoRI and BglII sites are underlined, the FLAG tag in small case, the stop codon in bold and sticky ends are in italic). Subsequently, the *E7GGG-His6* sequence was amplified with the same forward primer used to obtain the *E7GGG* gene, and the reverse primer 5′- ATAAAGATCTAGGTTTTTGTGAACAAATAGGA-3′ (the BglII site is underlined). The amplicon obtained was then digested with NdeI and BglII and cloned in the pCG2-FLAG vector obtaining the *E7GGG-FLAG* gene inserted between the *psbD* promoter/5′UTR and the *psbA* terminator/3′UTR.

### *Chlamydomonas* strains, transformation and growth conditions

For chloroplast transformation, the *C. reinhardtii* cell wall-less strain *cw15* was grown photomixotrophically until mid-log phase (concentration of 5×10^6^ cells/ml, measured using the TC10 automated cell counter, Bio-Rad) in TAP medium at 25°C under constant illumination of 120 µE m^−2^ s^−1^ on a rotary shaker. Cells were harvested by centrifugation and re-suspended in TAP medium to a final concentration of 3×10^8^ cells/ml. Chloroplast transformation was performed as previously described [35]. For each transformation, 300 µl of concentrated cells (about 10^8^ cells/ml) were agitated by vortexing for 20″ at 3,000 rpm in presence of 300 mg of glass beads (acid washed, Sigma, 425–600 µm) and 5 µg of plasmid DNA, and then spread onto TAP/agar plates containing 100 µg/ml spectinomycin. 50 transformants for each construct were propagated for 10 rounds in selective medium to obtain homoplasmic lines. For protein expression and characterization, transformants were grown in the same condition described for the *cw15* strain with the addition of spectinomycin 100 µg/ml in the culture media. To verify the correct integration in the chloroplast genome and the obtainment of the homoplasmic state, total DNA was extracted from all transformants with a standard phenol/chloroform extraction protocol. Correct integration and homoplasmy were checked by amplification with the primers indicated in Figure S2.

### Protein extraction and Western blot

For screening of transformants to identify the best expressor lines, 2×10^6^ *C. reinhardtii* cells were directly re-suspended in a suitable volume of loading buffer (10% glycerol, 60 mM Tris-HCl pH 6.8, 0.025% bromophenol blue, 2% SDS, 3% 2-mercaptoethanol) and boiled for 5′ before loading on a 12% SDS-PAGE gel. To analyze the solubility of the E7GGG protein variants cells were re-suspended in 1/20 culture volume of different buffers (described in Figure S4) and lysed on ice by sonication at 10 Hz output (3×10 seconds). *N. benthamiana* extracts were prepared by grinding the tissue to a fine powder in liquid nitrogen. The powder was re-suspended and homogenized with an ultraturrax in 3 volumes (w/v) of buffer containing protease inhibitors (“complete, EDTA-free”, Roche Diagnostics, GmbH, Mannheim, Germany). Soluble and insoluble proteins were separated by centrifugation for 20′ at 15,000 g at 4°C, with the resulting supernatant or insoluble pellet used in Western blot analysis. For all other experiments including characterization, purification, mice immunization, soluble proteins extracted using TS buffer (150 mM Tris-HCl, 200 mM sucrose, pH 7.5) were employed. Protein concentration was estimated using the Bradford assay (Bio-Rad Inc., Segrate, Italy). For Western blot analysis, proteins were transferred onto a PVDF membrane (GE Healthcare). After blocking with nonfat milk (5% in PBS), membranes were incubated 2 hours at R.T. with a 1∶3,000 dilution of a polyclonal anti-E7 antibody (sera of mice immunized with the purified E7-His6 protein produced in *E. coli*, kindly provided by Dr. P. Di Bonito, Istituto Superiore di Sanità, Rome). Membranes were then incubated for 1 hour at R.T. with a 1∶10,000 dilution of an anti-mouse peroxidase-conjugated secondary antibody (NA931, GE Healthcare) and the bound antibody was detected using the ECL Plus system (“Enhanced Chemi-Luminescence”, GE Healthcare). Protein quantification was performed by luminometry using a Chemidoc ImageLab system with ImageLab 4.0 software (Bio-Rad).

### E7GGG protein purification

Affinity purification of tagged, soluble E7GGG variants was performed in native conditions. The purification of the E7GGG-His6 protein was performed using the Ni-NTA affinity resin (Qiagen), while to purify the E7GGG-FLAG protein we used the anti-FLAG M2 Affinity gel (Sigma). In both cases, after optimization of purification conditions in small scale (100 ml of culture), we performed medium scale purifications (2–10 liters of culture) in order to accumulate protein for mice immunization. Briefly, 50 ml of soluble proteins in TS buffer extracted from 2 liters of culture were filtered (0.45 µm) and incubated with 1 ml of affinity resin for 2 hours. Resins were then washed with 200 ml of washing buffer and protein elution was obtained with 4–5 ml of elution buffer. E7GGG-His6 protein elution was performed with 50 mM NaH_2_PO_4_, 300 mM NaCl, 250 mM imidazole, pH 8.0. E7GGG-FLAG protein elution was performed with 1M Arg-HCl, pH 3.5. Purified proteins were then concentrated and dialyzed against PBS+0.1 mM ZnSO_4_ using Amicon Ultra-4 (Millipore) dialysis tubes with a 3 kDa cut-off.

### Mice immunization, evaluation of immune responses and tumor challenge

Four-week-old female C57BL/6 mice (Charles Rivers, Como, Italy) were used. Mice were maintained under specific pathogen-free conditions at the Experimental Animal Department of the Regina Elena National Cancer Institute (Rome, Italy). Groups of 8 mice were vaccinated subcutaneously on days 0, 14, 28, 42, and 56 with the following preparations (all in PBS+0.1 mM ZnSO_4_) 1) 200 µl of buffer; 2) 500 µl of soluble extract from wt *Chlamydomonas*; 3) 500 µl of soluble extract from a *Chlamydomonas* transformant containing about 1 µg of the E7GGG protein); 4) 200 µl with 2 µg of purified E7GGG-FLAG protein; 5) 200 µl with 2 µg of purified *E. coli* E7GGG-His6 protein. Adjuvant QuilA (10 µg/mouse) was added to all vaccine preparations. One week after the first and the last boost, all animals were subjected to ELISA and spontaneous Delayed-Type Hypersensitivity (DTH) assays, and after the last boost three animals in each group were sacrificed to evaluated cell-mediated immune responses by Enzyme-Linked Immunosorbent Spot (ELISPOT) analysis. All remaining animals were then challenged by sub-cutaneous injection of 5×10^4^ E7-expressing TC-1 tumor cells [41].

Sera of mice collected one week after the last boosts were analyzed by ELISA assay for the presence of E7-specific antibodies. Microtiter plates were coated with 100 ng/well of E7-His6 protein from *E. coli* diluted in PBS buffer (pH 7.2) or bicarbonate buffer (50 mM NaHCO_3_, pH 9.6). Serial dilution of sera in PBS+2% non-fat milk were added to the coated wells, followed by anti-mouse peroxidase-conjugated secondary antibody (NA931, GE Healthcare) diluted 1∶10,000. Colorimetric reaction was induced by adding 100 µl/well v/v H_2_O_2_/ABTS [2′, 2′-azino bis-(3-etilbenzotiazolin) sulphuric acid] (KPL Inc., Gaithersburg, MD-USA). The ELISA antibody titers were calculated as the log_10_ of the reciprocal antibody dilution that showed an OD_405_ value above the cut-off value, which was defined as the average OD_405_ value of non-immunized sera+3 standard deviations. DTH was performed as previously described [27]. Ear thickness was assessed 48 and 72 h after challenge using a microcaliper and ear swelling was reported as the difference between the challenged and the unchallenged control ear. HPV16 E7-specific T-cell precursors were detected by ELISPOT as previously described [27]. Briefly, single cell suspension of splenocytes (10^6^ cells/well), harvested from three vaccinated mice/group, was added to microtiter wells coated with a rat anti-mouse IFN-γ antibody (5 µg/ml; clone R4-6A2, BD Bioscience PharMingen, San Diego, CA, USA) along with interleukin 2 (50 units/ml; Sigma-Aldrich, St. Louis, Missouri, USA). Samples were incubated with or without 1 µg/ml of E7-specific H-2Db CTL epitope (aa 49–57, RAHYNIVTF) [47] at 37°C for 24 h. Plates were then incubated with an anti-IFN-γ biotinylated antibody (2 µg/ml; clone XMG1.2, BD Bioscience PharMingen, San Diego, CA, USA) followed by Streptavidin-HRP (2.5 µg/ml, BD Bioscience PharMingen, San Diego, CA, USA). Spots were developed by adding 3.3′-diaminobenzide/peroxidase substrate Sigma Fast (Sigma-Aldrich St. Louis, Missouri, USA) and counted using a dissecting microscope.

## Supporting Information

Figure S1 **Sequence of the** ***Chlamydomonas*** **cassette expressing the E7GGG protein variants.** The sequence of the *E7GGG* gene is in red, with the start and stop codons in bold. The *E7GGG-His6* gene variant contains the additional sequence highlighted in blue, that comprises the His6-tag and the thrombin site. The *E7GGG-FLAG* gene variant contains the additional sequence highlighted in green that comprises the FLAG-tag. Restriction sites are underlined.
(TIF)

Figure S2 **Verification of transgene integration and confirmation of homoplasmy.** **A.** Integration scheme of the pCG2 plasmid. The integration occurs in *psaA* intron between nucleotides 158321–160126 (acc. NC_005353.1). The 5′ flanking region (5′ fl.) comprises nucleotides 157103–158321; the 3′ flanking region (3′ fl.) comprises nucleotides 160126–162410. Triangles of different colors joined by horizontal lines indicate primers used to verify correct integration and homoplasmy, and the relative amplicons. Amplicons 1 and 2 indicate correct integration, amplicon 3 indicates lack of homoplasmy, amplicon 4 is a positive control for the presence of chloroplast DNA. **B.** Sequences of the primers. **C.** PCR results of four representative transformants (a–d) after 10 rounds of restreaking on selective medium: the presence of amplicons 1 and 2 confirms the correct integration, the absence of amplicon 3 indicates that the lines are homoplasmic for the presence of the transgene.
(TIF)

Figure S3 **Growth curves of the best expressors for each protein variant.** Cell concentration (cells/ml) was measured at 0, 12, 24 and 48 hours. Control = transformant obtained with the pCG1 vector. Error bars represent standard deviation of three biological replicates.
(TIF)

Figure S4 **Comparison of the solubility of the E7GGG protein produced in** ***C. reinhardtii*** **by chloroplast transformation or in** ***N. benthamiana*** **plants by PVX-mediated infection.** Immunoblotting of soluble fraction (S) and insoluble pellet (I) (5 µl each = 20 µg of total proteins in the soluble fraction) from *Chlamydomonas* and *N. benthamiana* extracted using the following buffers: (1) 100 mM HEPES-KOH pH 5.0, 200 mM sucrose; (2) 100 mM HEPES-KOH pH 6.0, 200 mM sucrose; (3) 100 mM Tris-HCl pH 7.0, 200 mM sucrose; (4) 100 mM Tris-HCl pH 8.0, 200 mM sucrose; 5) 100 mM Tris-HCl pH 7.0, 154 mM NaCl; (6) 100 mM Tris-HCl pH 7.0, 200 mM sucrose, 1 mM Triton X-100; (7) PBS (21 mM Na_2_HPO_4_, 2.1 mM NaH_2_PO_4_, 150 mM NaCl, pH 7.2).
(TIF)

Figure S5 **Optimization of E7GGG-FLAG affinity purification.** Western of 10 µl of the following samples: lane 1: E7GGG-FLAG extract before purification; lane 2: flow-through; lanes 3, 4: elution with 0.1 M glycine pH 3.5; lanes 5, 6: elution with 0.1 M glycine pH 2.5; lanes 7, 8: elution with 1 M Arg-HCl pH 3.5; lanes 9, 10: elution with 100 µg/ml FLAG peptide; lane 11: empty resin (negative control). e = eluted fraction r = resin after protein elution.
(TIF)

Figure S6 **E7GGG-His6 protein characterization.** **A.** Western blot of 20 µg of TSP extracted from the E7GGG-His6 transformant and treated as follows: lane 1: boiling for 5′ in presence of 10 mM 2-mercaptoethanol; lane 2: boiling for 10′ in presence of 10 mM 2-mercaptoethanol; lane 3: boiling for 5′ in presence of 10 mM 2-mercaptoethanol and 10 mM DTT; lane 4: boiling for 10′ in presence of 10 mM 2-mercaptoethanol and 10 mM DTT; lanes 5, 6: purified E7GGG-His6 protein from *E. coli* 2 and 5 ng, respectively. **B.** Western blot after calf intestinal phosphatase (CIP) treatment of 20 µg TSP at 37°C. Lane 1: 40 U CIP 30′; lane 2: 40 U CIP 60′; lane 3: untreated 30′; lane 4: untreated 60′.
(TIF)
