# Supplementary figures and images for: A Chlamydomonas-Derived Human Papillomavirus 16 E7 Vaccine Induces Specific Tumor Protection
Source: PLoS One. 2013 Apr 23;8(4):e61473. doi: 10.1371/journal.pone.0061473 (PMC3634004; doi:10.1371/journal.pone.0061473)

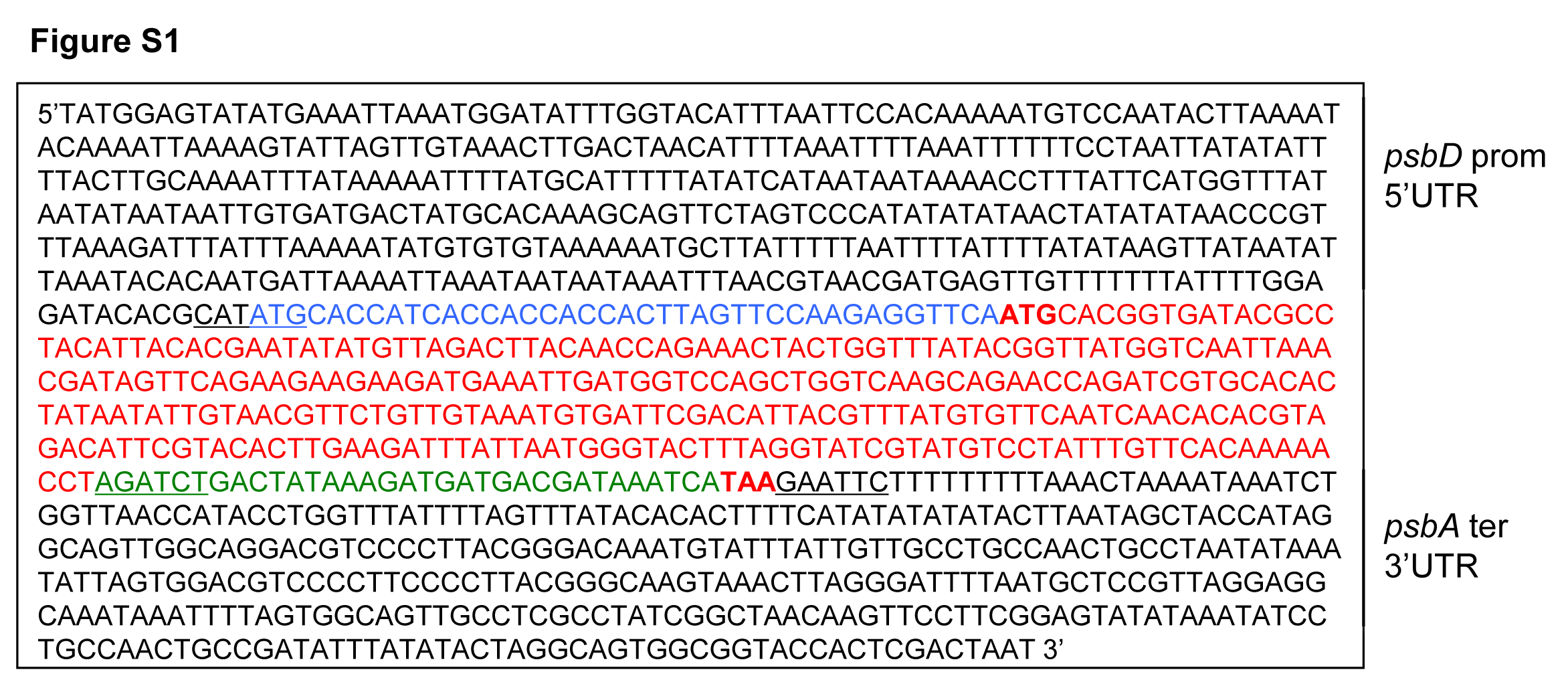

Supplement: Figure S1 — Sequence of the Chlamydomonas cassette expressing the E7GGG protein variants. The sequence of the E7GGG gene is in red, with the start and stop codons in bold. The E7GGG-His6 gene variant contains the additional sequence highlighted in blue, that comprises the His6-tag and the thrombin site. The E7GGG-FLAG gene variant contains the additional sequence highlighted in green that comprises the FLAG-tag. Restriction sites are underlined. (TIF) [file pone.0061473.s001.tif]

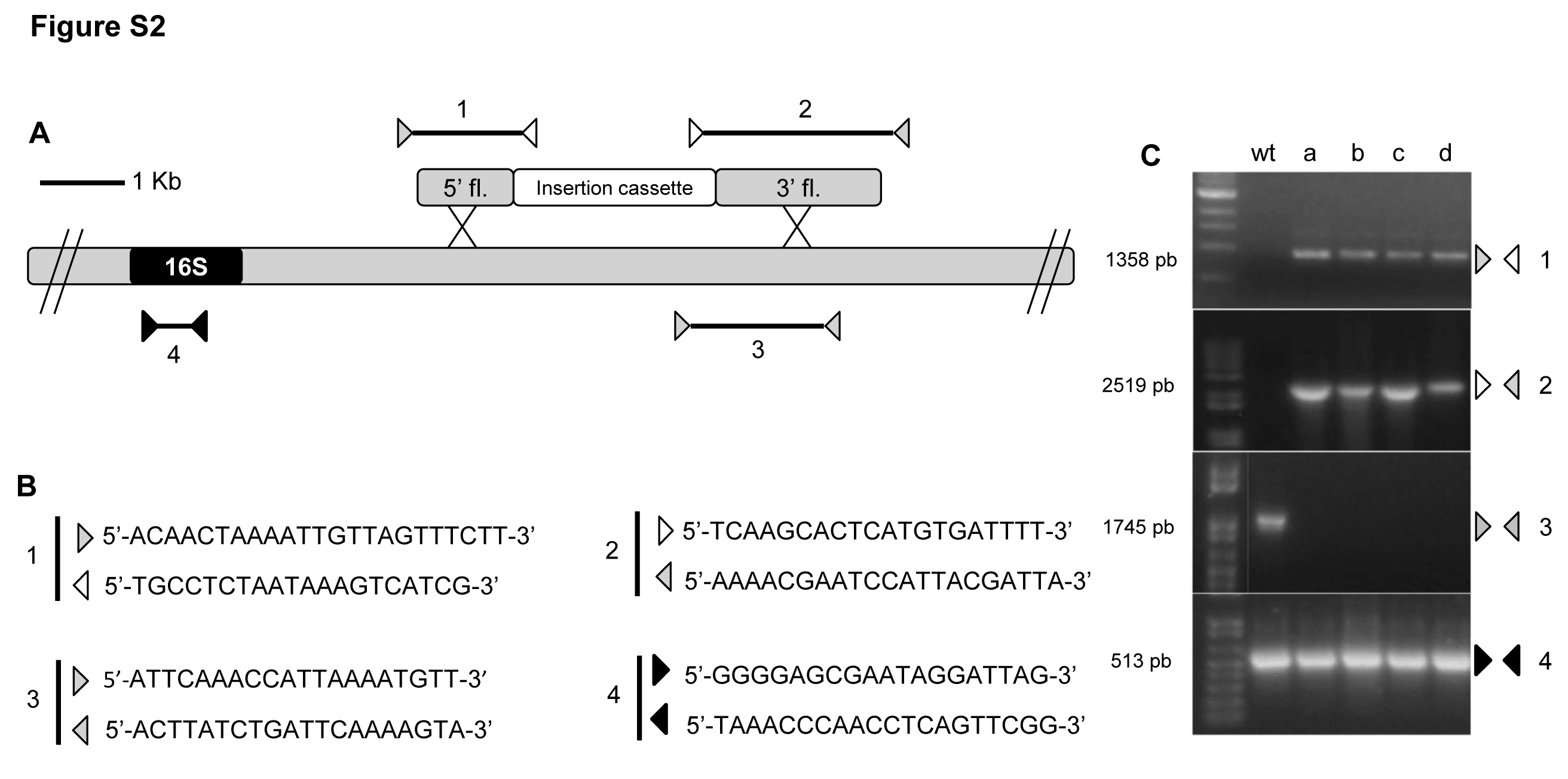

Supplement: Figure S2 — Verification of transgene integration and confirmation of homoplasmy. A. Integration scheme of the pCG2 plasmid. The integration occurs in psaA intron between nucleotides 158321–160126 (acc. NC_005353.1). The 5′ flanking region (5′ fl.) comprises nucleotides 157103–158321; the 3′ flanking region (3′ fl.) comprises nucleotides 160126–162410. Triangles of different colors joined by horizontal lines indicate primers used to verify correct integration and homoplasmy, and the relative amplicons. Amplicons 1 and 2 indicate correct integration, amplicon 3 indicates lack of homoplasmy, amplicon 4 is a positive control for the presence of chloroplast DNA. B. Sequences of the primers. C. PCR results of four representative transformants (a–d) after 10 rounds of restreaking on selective medium: the presence of amplicons 1 and 2 confirms the correct integration, the absence of amplicon 3 indicates that the lines are homoplasmic for the presence of the transgene. (TIF) [file pone.0061473.s002.tif]

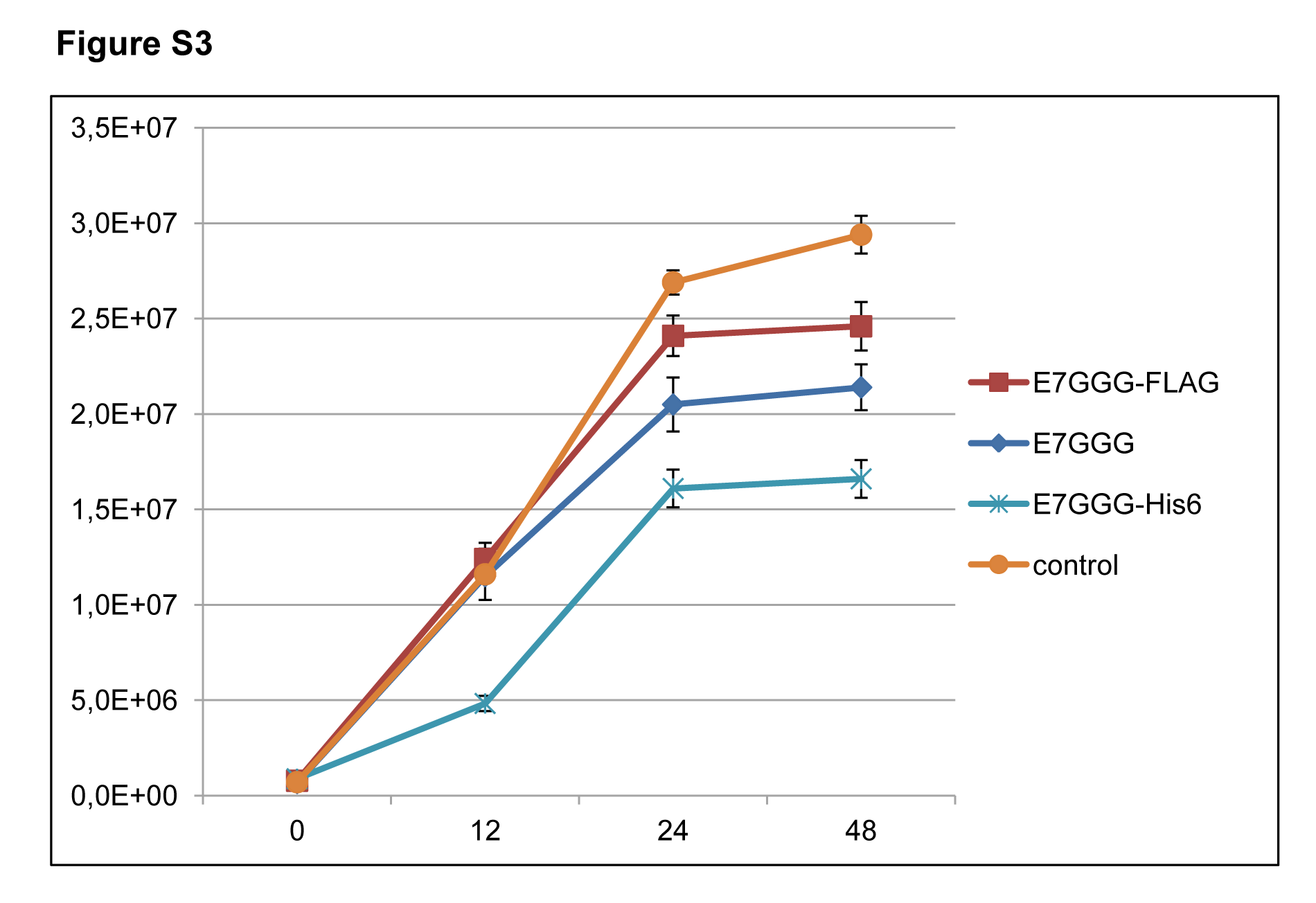

Supplement: Figure S3 — Growth curves of the best expressors for each protein variant. Cell concentration (cells/ml) was measured at 0, 12, 24 and 48 hours. Control = transformant obtained with the pCG1 vector. Error bars represent standard deviation of three biological replicates. (TIF) [file pone.0061473.s003.tif]

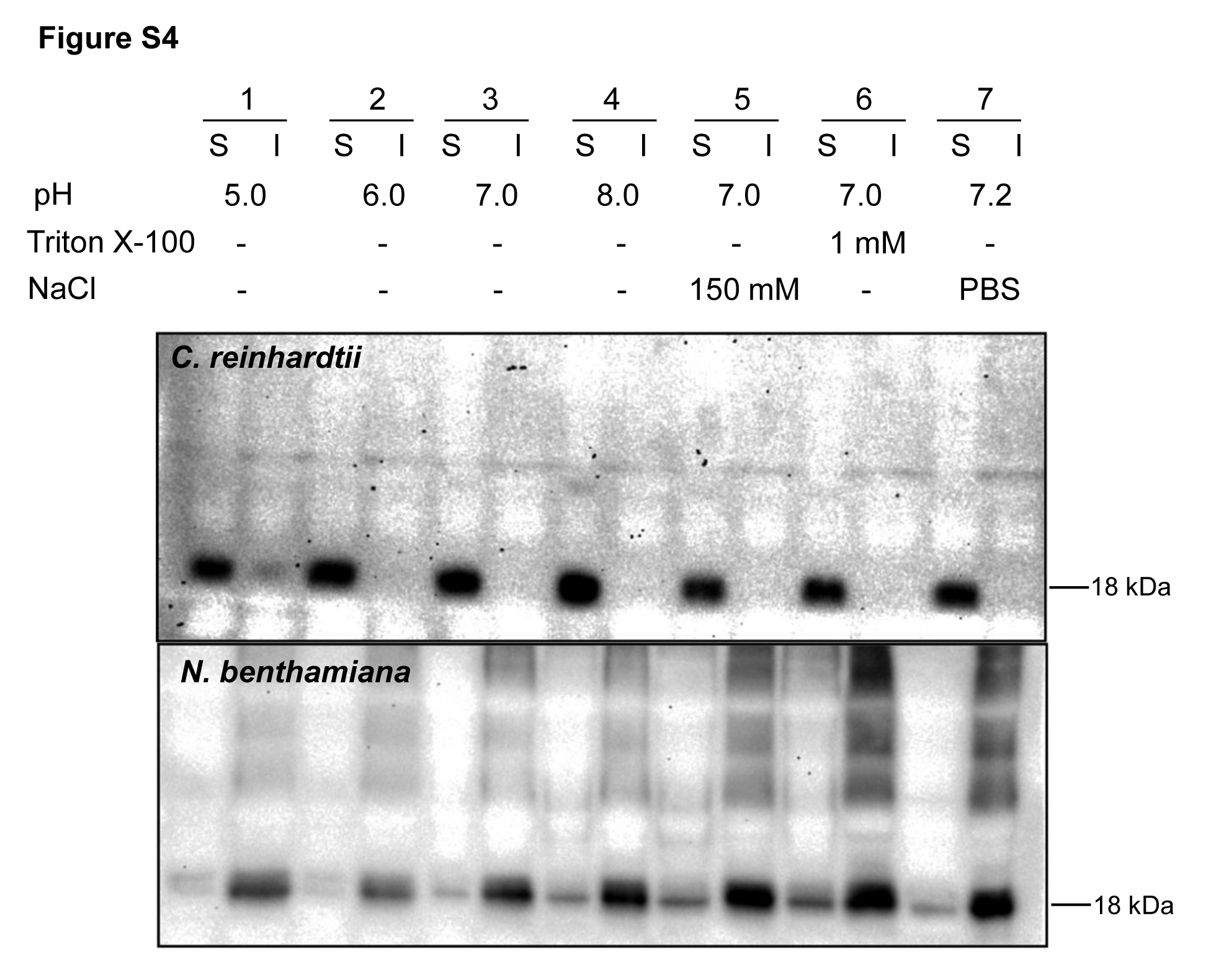

Supplement: Figure S4 — Comparison of the solubility of the E7GGG protein produced in C. reinhardtii by chloroplast transformation or in N. benthamiana plants by PVX-mediated infection. Immunoblotting of soluble fraction (S) and insoluble pellet (I) (5 µl each = 20 µg of total proteins in the soluble fraction) from Chlamydomonas and N. benthamiana extracted using the following buffers: (1) 100 mM HEPES-KOH pH 5.0, 200 mM sucrose; (2) 100 mM HEPES-KOH pH 6.0, 200 mM sucrose; (3) 100 mM Tris-HCl pH 7.0, 200 mM sucrose; (4) 100 mM Tris-HCl pH 8.0, 200 mM sucrose; 5) 100 mM Tris-HCl pH 7.0, 154 mM NaCl; (6) 100 mM Tris-HCl pH 7.0, 200 mM sucrose, 1 mM Triton X-100; (7) PBS (21 mM Na2HPO4, 2.1 mM NaH2PO4, 150 mM NaCl, pH 7.2). (TIF) [file pone.0061473.s004.tif]

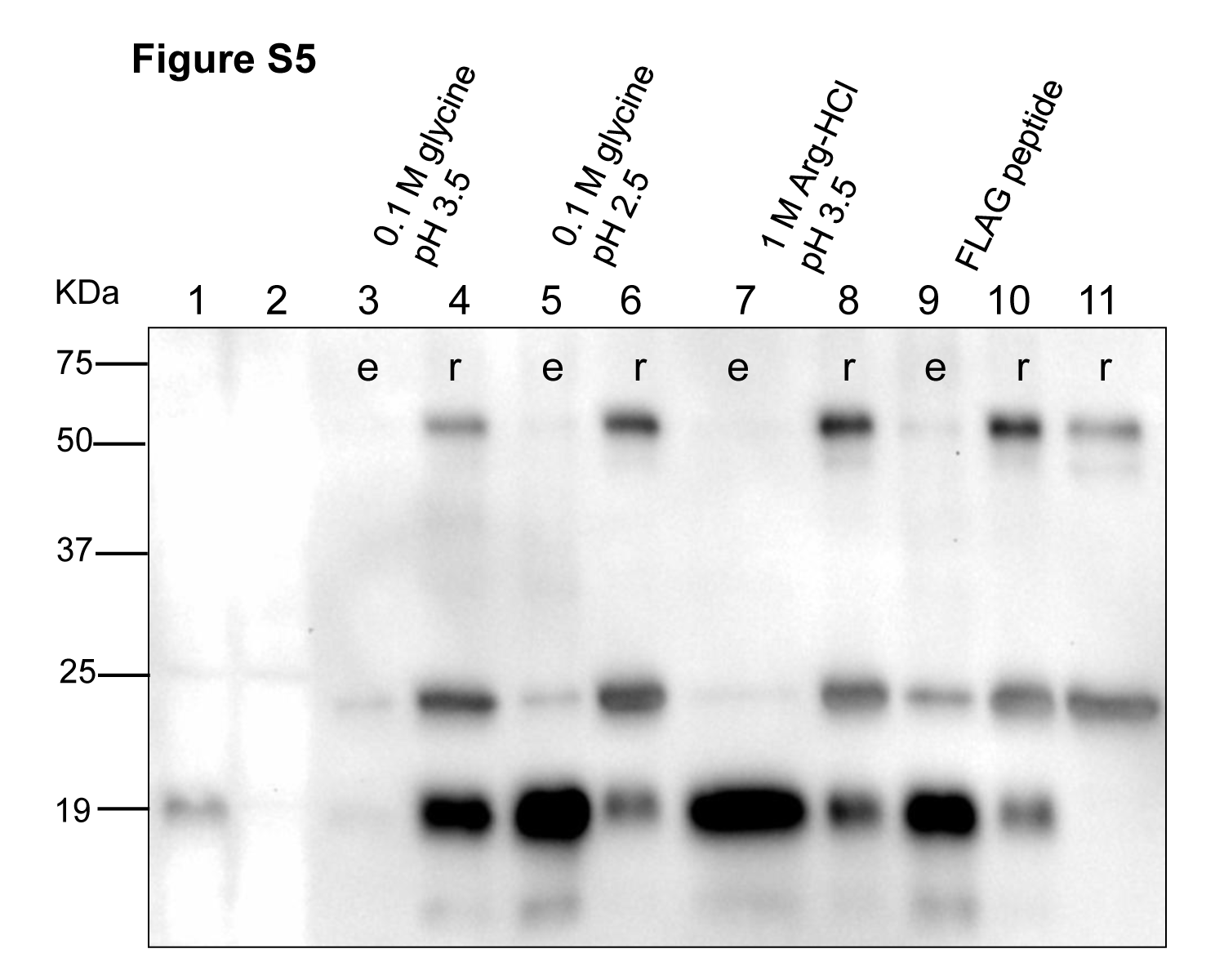

Supplement: Figure S5 — Optimization of E7GGG-FLAG affinity purification. Western of 10 µl of the following samples: lane 1: E7GGG-FLAG extract before purification; lane 2: flow-through; lanes 3, 4: elution with 0.1 M glycine pH 3.5; lanes 5, 6: elution with 0.1 M glycine pH 2.5; lanes 7, 8: elution with 1 M Arg-HCl pH 3.5; lanes 9, 10: elution with 100 µg/ml FLAG peptide; lane 11: empty resin (negative control). e = eluted fraction r = resin after protein elution. (TIF) [file pone.0061473.s005.tif]

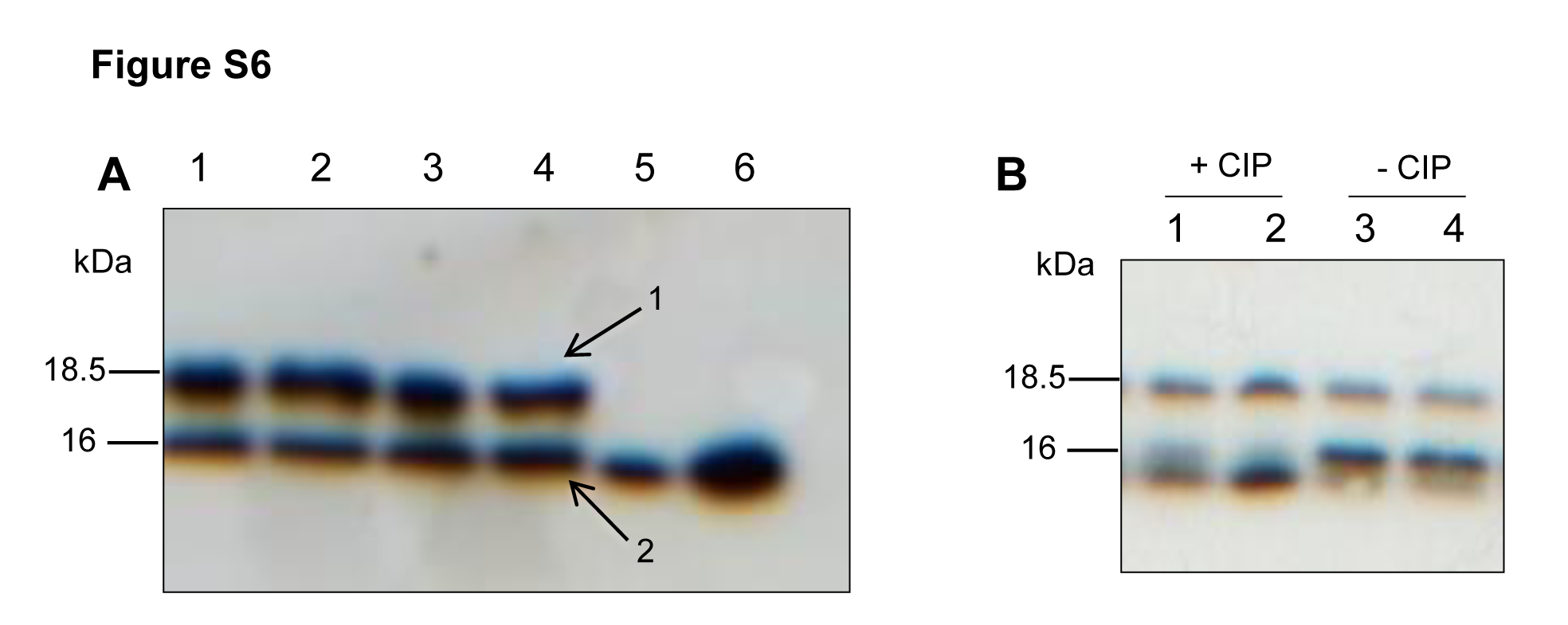

Supplement: Figure S6 — E7GGG-His6 protein characterization. A. Western blot of 20 µg of TSP extracted from the E7GGG-His6 transformant and treated as follows: lane 1: boiling for 5′ in presence of 10 mM 2-mercaptoethanol; lane 2: boiling for 10′ in presence of 10 mM 2-mercaptoethanol; lane 3: boiling for 5′ in presence of 10 mM 2-mercaptoethanol and 10 mM DTT; lane 4: boiling for 10′ in presence of 10 mM 2-mercaptoethanol and 10 mM DTT; lanes 5, 6: purified E7GGG-His6 protein from E. coli 2 and 5 ng, respectively. B. Western blot after calf intestinal phosphatase (CIP) treatment of 20 µg TSP at 37°C. Lane 1: 40 U CIP 30′; lane 2: 40 U CIP 60′; lane 3: untreated 30′; lane 4: untreated 60′. (TIF) [file pone.0061473.s006.tif]
